# The Role of Monocyte to High-Density Lipoprotein Cholesterol Ratio in Prediction of Carotid Intima-Media Thickness in Patients With Type 2 Diabetes

**DOI:** 10.3389/fendo.2019.00191

**Published:** 2019-04-04

**Authors:** Jia Wei Chen, Chang Li, Zhu Hui Liu, Ying Shen, Feng Hua Ding, Xin Yi Shu, Rui Yan Zhang, Wei Feng Shen, Lin Lu, Xiao Qun Wang

**Affiliations:** ^1^Department of Cardiology, Rui Jin Hospital, Shanghai Jiao Tong University School of Medicine, Shanghai, China; ^2^Institute of Cardiovascular Diseases, Shanghai Jiao Tong University School of Medicine, Shanghai, China

**Keywords:** monocyte to high-density lipoprotein cholesterol ratio, carotid intima-media thickness, atherosclerosis, type 2 diabetes, subclinical carotid atherosclerosis

## Abstract

**Background:** Chronic inflammatory disorders and dyslipidemia in type 2 diabetes mellitus (T2DM) are essential contributors to the development of atherosclerotic cardiovascular disease. Monocyte to high-density lipoprotein cholesterol (HDL-C) ratio (MHR) is a novel and simple measure associated positively with the body inflammatory and oxidative stress status. However, little is known regarding the role of MHR in evaluating carotid intima-media thickness (CIMT), a surrogate predictor of subsequent vascular events, especially in diabetic patients.

**Methods:** A total of 494 patients with T2DM and 1,848 non-diabetic subjects were consecutively enrolled in study 1. Correlation between MHR and CIMT was compared between diabetic and non-diabetic subjects. In study 2, a total of 110 T2DM patients from study 1 with normal basal CIMT and a follow-up ultrasonography at 12 months were enrolled. The predictive role of MHR on CIMT progression in diabetic patients was analyzed.

**Results:** In study 1, MHR was higher in patients with T2DM than non-diabetic subjects (*p* < 0.001). After adjustment for confounding risk factors, MHR remained correlated significantly with CIMT in diabetic (*r* = 0.172, *p* = 0.001) but not non-diabetic (*r* = 0.006, *p* = 0.813) subjects. Logistic regression analyses demonstrated that MHR is superior to traditional lipid parameters in association with elevated CIMT in diabetic patients. In study 2, MHR at baseline was positively correlated with change in CIMT (*r* = 0.313, *p* = 0.001). Basal MHR was independently associated with change in CIMT [β = 0.059, (95% CI: 0.012–0.105), *p* = 0.014] in multivariate linear regression analysis.

**Conclusions:** Our study suggests that MHR is a convenient and effective measure in prediction of the presence and progression of subclinical carotid atherosclerosis in patients with T2DM.

## Introduction

Patients with type 2 diabetes mellitus (T2DM) are predisposed to develop atherosclerosis, which is largely attributable to the disturbed glucose and lipid metabolism as well as the chronic inflammatory status (1, 2). The compositional changes of lipoprotein particles in diabetic conditions, as characterized by the formation of atherogenic small dense low-density lipoprotein (LDL) particles and the predominance of large very-low density lipoprotein (VLDL) particles, usually lead to underappreciation of the risk associated with the atherogenic lipoprotein by simply measuring the cholesterol content in LDL (LDL-C) (3). On the other hand, chronic inflammation plays a comparable role in driving atherosclerosis in diabetic conditions (4, 5). Monocyte is one of the circulating makers of systemic inflammation and a fundamental player in atherogenesis (6). The entire process of monocytosis, adhesion, and infiltration of monocytes to the vessel wall, and the subsequent transformation into lipid-laden macrophages has been well-described in human and animal studies (7, 8). Several lines of evidence have shown that count of monocytes or monocyte subsets are independent predictors of subclinical atherosclerosis or coronary artery disease (9, 10).

Monocyte to HDL cholesterol ratio (MHR) was recently defined as a novel marker in relation to the extent of inflammation and oxidative stress as well as adverse cardiovascular outcomes (11). Previous reports have shown that MHR is associated with cardiovascular events in patients with chronic kidney disease (12), and in-hospital and long-term death in patients with infective endocarditis and normal left ventricular function (13).

Carotid intima-media thickness (CIMT) is a simple and cost-effective surrogate phenotype of subclinical atherosclerosis. A number of longitudinal studies have evidenced the predictive value of CIMT for subsequent cardiac and cerebrovascular events (14, 15). In the present study, we investigated the association of MHR with CIMT in diabetic and non-diabetic subjects. We also analyzed the role of MHR in prediction of CIMT progression by performing carotid ultrasonography at 1 year follow-up in T2DM patients.

## Methods

### Study Population

This study complies with the Declaration of Helsinki. The study protocol was approved by the local hospital ethics committee, and written informed consent was obtained from all participants.

**Figure 1:**
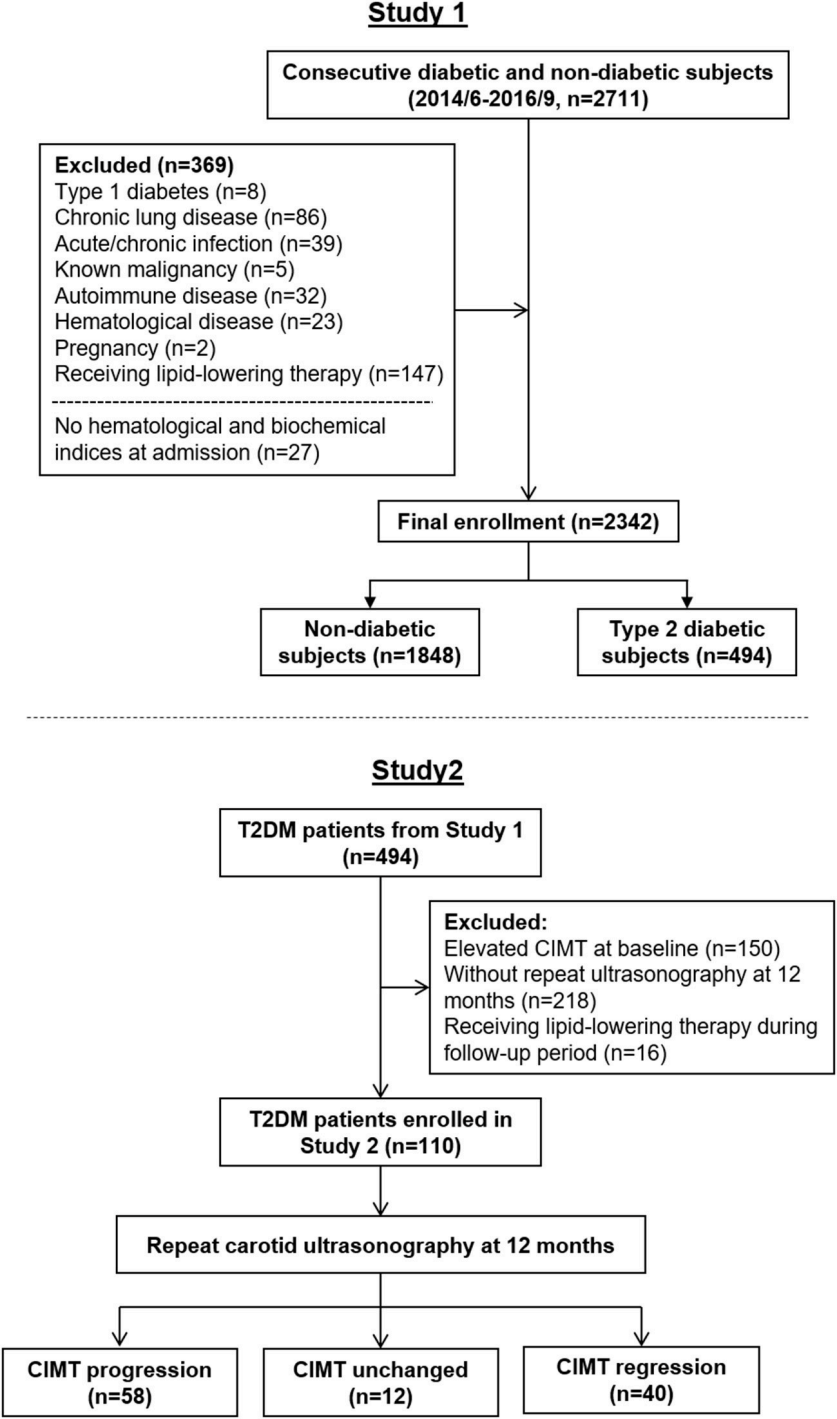
Flow charts of patient enrollment in Study 1 and Study 2.

For the purpose of this study to assess preclinical atherosclerosis, we consecutively enrolled 2,711 subjects in study 1 (Figure 1, upper part) based on the following criteria (1) aged between 35 and 80 years old (2), no manifest concomitant atherosclerotic disease, from June, 2014 to September, 2016 in the Department of Cardiology, Rui Jin Hospital, Shanghai Jiao Tong University School of Medicine. To avoid confounding data, we excluded 342 patients due to type 1 diabetes, pregnancy, chronic lung disease, chronic or acute infection, known malignancy, autoimmune or hematologic disease, or receiving lipid-lowering therapy. Another 27 patients who did not have hematological and biochemical indices that included monocyte and HDL-C on admission were also excluded. Thus, 2,342 patients comprised the final enrollment. The diagnosis of diabetes was made according to the criteria of American Diabetes Association (16). Hypertension and dyslipidemia were diagnosed according to seventh report of the Joint National Committee on prevention, detection, evaluation, and treatment of high blood pressure (JNC 7) and guideline of the National Cholesterol Education Program (ATP III), respectively (17, 18).

To investigate the predictive role of MHR on CIMT progression in T2DM patients with relatively normal CIMT, a total of 126 diabetic patients from study 1 with normal CIMT (within the first three quartiles, <0.73 mm) and a follow-up ultrasonography at 12 months were enrolled in study 2 (Figure 1, bottom part). Sixteen patients were excluded due to receiving lipid-lowering therapy within this period. The change in CIMT per year (mm/year) was calculated. The association between change in CIMT and MHR was then analyzed in the final 110 diabetic patients.

### Clinical and Biochemical Assessments

Blood samples were collected after an overnight fasting. Serum glucose, blood urea nitrogen, creatinine, uric acid, total cholesterol, low-density lipoprotein-cholesterol (LDL-C), high-density lipoprotein cholesterol (HDL-C), and triglycerides were assessed (HITACHI 912 Analyzer, Roche Diagnostics, Germany). The estimated glomerular filtration rate (eGFR) was computed using the Chronic Kidney Disease Epidemiology Collaboration equation (19). Blood HbA1c concentration was measured using ion-exchange high performance liquid chromatography with Bio-rad Variant Hemoglobin Testing System (Bio-Rad Laboratories, USA). Serum levels of high sensitive C-reactive protein (hsCRP) were determined by ELISA (Biocheck Laboratories, Toledo, OH, USA). The detailed information about medical history and lifestyles including smoking and drinking status was obtained using a standard questionnaire by the trained physicians. Current smoking status was defined as yes if the subject smoked at least one cigarette per day or seven cigarettes per week in the past 6 months. Body mass index (BMI) was calculated using the formula of weight/height^2^ (kilograms per square meter). Blood pressure was measured on the non-dominant arm in a seated position after a 10-min rest, using an electronic blood pressure monitor (OMRON Model HEM-752 FUZZY' Omron Co., Dalian, China). Three measurements were taken at 1-min intervals, and the average was used for analysis. MHR was calculated by monocyte counts (× 10^6^/L)/HDL-C (mg/dL).

### CIMT Measurements

CIMT measurements were performed manually using a high-resolution B-mode tomographic ultrasound system (Esaote Mylab90, Italy) with a linear 10 MHz transducer. Precision of the CIMT measurement is 0.01 mm. The sonographers measured CIMT on the far-wall of the right and left common carotid arteries, 1.5 cm proximal to the bifurcation. The transducer was manipulated so that the lumen diameter was maximized in the longitudinal plane. The first and second lines represent the lumen–intimal interface and the collage-contained upper layer of tunic adventitia, respectively. The mean value of the right and left common carotid IMT was used for analysis. The fourth quartile of CIMT (≥0.73 mm) was defined as elevated CIMT. In study 2, the baseline and follow-up CIMT were recorded as CIMT1 and CIMT2, respectively. Progression of CIMT was calculated as the difference between CIMT1 and CIMT2 and recorded as change in CIMT. The coefficient of variations was <5.1%.

### Statistics

Continuous variables were presented as mean (SD or SEM), and categorical data were summarized as frequencies (percentages). For categorical clinical variables, differences between groups were evaluated by the chi-square test followed by Bonferroni's correction. For continuous variables, normal distribution was evaluated with Kolmogorov–Smirnov test, and logarithmic transformations were performed on the continuous variables of non-normal distribution. Differences among groups were analyzed by Student's *t*-test or one-way analysis of variance (ANOVA) followed by *post-hoc* Bonferroni test. Correlation between variables was determined by Pearson's correlation test. In study 1, different logistic regression models were implemented to interrogate the association of different lipid parameters with elevated CIMT in patients with T2DM and non-diabetic patients. In model 1, no covariates were adjusted; in model 2, age, sex, history of hypertension and smoking were adjusted; in model 3, eGFR, logarithmically transformed levels of high sensitive C-reactive protein, HbA1c, and triglyceride were further adjusted. In study 2, linear regression was performed to evaluate the associations between the change in CIMT and MHR in patients with T2DM. Confounders adjusted in the linear regression included baseline CIMT, age, sex, history of hypertension and smoking, logarithmically transformed levels of HbA1c, and triglyceride. All statistical analyses were performed using the SPSS 23.0 for Windows (SPSS, Inc., Chicago, IL, USA). A two-tailed <0.05 was considered statistically significant.

## Results

### Study 1

#### Characteristics of the Studied Population

A total of 494 patients with T2DM and 1,848 non-diabetic subjects were analyzed in study 1 (Table 1). Compared to non-diabetic subjects, patients with T2DM were of older age and had higher prevalence of hypertension. Counts of total white blood cells, neutrophils, lymphocytes, and levels of high sensitivity C-reactive protein (hsCRP), triglyceride were higher, whereas levels of HDL-C and apolipoprotein A-I (apoA-I) were lower in diabetic than non-diabetic patients. No significant difference in levels of total cholesterol, LDL-C, serum creatine, and blood urea nitrogen was detected between two groups. The monocyte to HDL-C ratio (MHR) was higher in T2DM patients than non-diabetic subjects [10.92 (interquartile rage (IQR): 8.34–14.02) vs. 9.95 (IQR: 7.32–12.93), *p* < 0.001].

**Table 1:** Baseline characteristics of diabetic and non-diabetic subjects.

	**non-DM**	**DM**	***P*-value**
Number	1,848	494	
Male gender, *n* (%)	958 (51.8)	276 (55.9)	0.116
Age, years	59.73 ± 8.89	60.71 ± 9.07	0.030
Body mass index, kg/m^2^	24.64 ± 3.38	25.49 ± 3.71	<0.001
Smoking, *n* (%)	344 (18.6)	94 (19.0)	0.845
Hypertension, *n* (%)	942 (51.0)	329 (66.6)	<0.001
Systolic blood pressure, mmHg	132.28 ± 17.93	136.57 ± 18.70	<0.001
Diastolic blood pressure, mmHg	76.95 ± 11.08	76.82 ± 11.52	0.813
White blood cells (10^9^/mL)	5.88 ± 1.40	6.27 ± 1.62	<0.001
Neutrophils (10^9^/mL)	3.37 ± 1.10	3.60 ± 1.21	<0.001
Lymphocytes (10^9^/mL)	1.87 ± 0.58	1.99 ± 0.60	<0.001
Monocytes (10^9^/mL)	0.46 ± 0.14	0.47 ± 0.14	0.254
Platelets (10^9^/mL)	183.98 ± 49.68	184.66 ± 51.45	0.792
hsCRP, mg/L	0.73 (0.40–1.52)	0.90 (0.46–1.93)	0.002
Serum creatinine, μmol/L	72.53 ± 12.12	72.71 ± 14.21	0.788
Blood urea nitrogen, mmol/L	5.48 ± 1.37	5.58 ± 1.40	0.164
Uric acid, μmol/L	327.50 ± 81.23	319.37 ± 81.33	0.049
eGFR, mL/min/1.73 m^2^	111.67 ± 17.53	114.85 ± 21.14	0.001
HbA1c, %	5.60 (5.40–5.90)	6.90 (6.30–7.80)	<0.001
Fasting glucose, mmol/L	4.97 (4.58–5.42)	6.43 (5.47–7.77)	<0.001
Postprandial glucose (2 h), mmol/L	6.55 (5.56–7.61)	13.43 (10.36–16.52)	<0.001
Fasting insulin, μIU/mL	8.33 (5.75–11.59)	9.49 (6.30–14.65)	<0.001
Postprandial insulin (2 h), μIU/mL	46.76 (25.29–77.26)	47.34 (27.58–79.11)	0.680
Triglyceride, mmol/L	1.30 (0.97–1.83)	1.58 (1.10–2.21)	<0.001
Total cholesterol, mmol/L	4.33 ± 1.01	4.32 ± 1.14	0.766
HDL cholesterol, mmol/L	1.21 ± 0.29	1.12 ± 0.29	<0.001
LDL cholesterol, mmol/L	2.58 ± 0.82	2.55 ± 0.90	0.471
Apolipoprotein A-I, g/L	1.31 ± 0.20	1.28 ± 0.22	0.004
Apolipoprotein B, g/L	0.82 ± 0.22	0.85 ± 0.24	0.052
MHR	9.95 (7.32–12.93)	10.92 (8.34–14.02)	<0.001
Oral hypoglycemic drugs, *n* (%)	–	338 (68.4)	–
Insulin, *n* (%)	–	131 (26.5)	–

#### Correlation Analyses of CIMT and MHR

**Figure 2:**
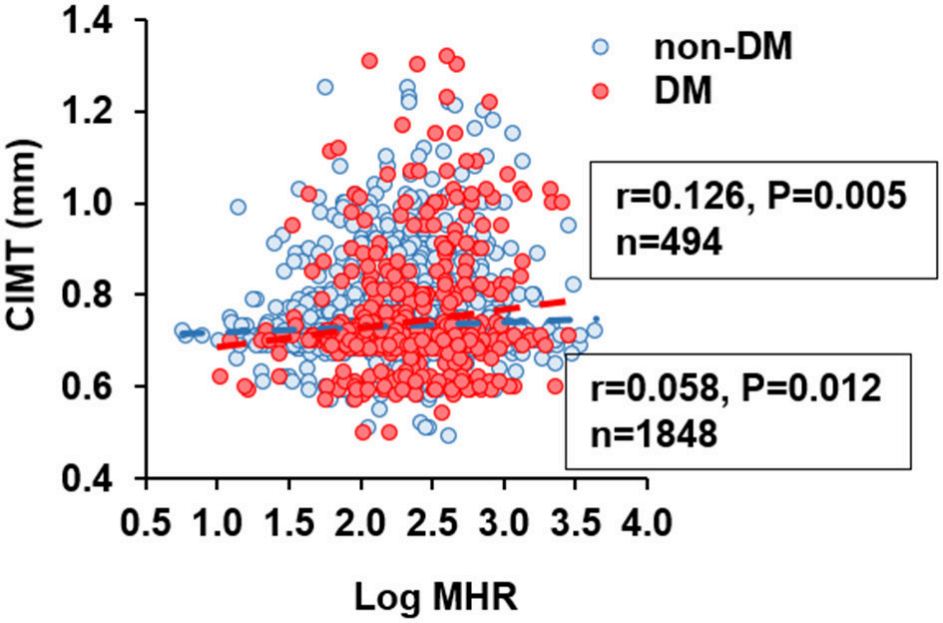
Correlation between carotid intima-media thickness (CIMT) and monocyte to HDL cholesterol ratio (MHR). MHR was logarithmically transformed before plotting. Blue circle and dashed line, non-diabetic subjects (*n* = 1848); red circle and dashed line, patients with type 2 diabetes (*n* = 494).

**Figure 3:**
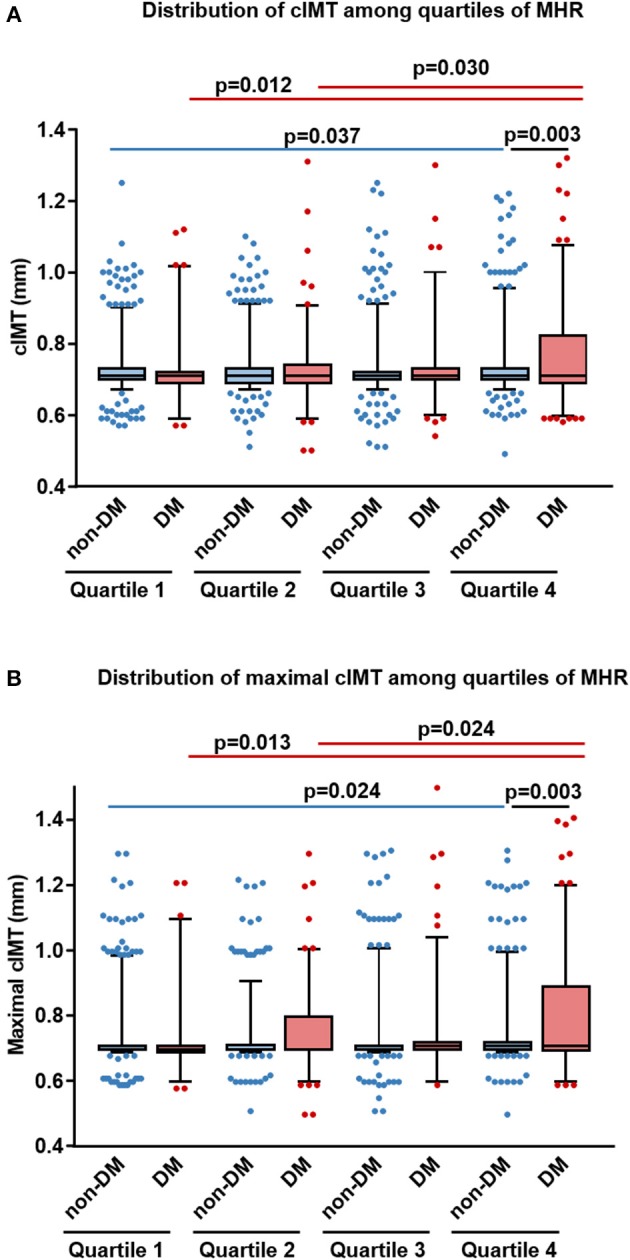
Distribution of average and maximal carotid intima-media thickness (CIMT) among different quartiles of monocyte to HDL cholesterol ratio (MHR) in diabetic and non-diabetic subjects. Box and whisker plots of the average **(A)** and maximal **(B)** CIMT in different quartiles of MHR were shown (horizontal bars in box: low, 25 percentile; middle, median; upper, 75 percentile; low whisker, 5 percentile, and upper whisker, 95 percentile). DM, patients with type 2 diabetes; non-DM, non-diabetic patients.

In non-diabetic subjects, we found CIMT was correlated positively with age, systolic blood pressure (BP), BMI, LDL-C, non-HDL cholesterol, apolipoprotein (apoB), log-transformed levels of hsCRP, HbA1C, and MHR (*r* = 0.058, *p* = 0.012), while negatively with HDL-C and apoA-I. However, most of these associations were attenuated in patients with T2DM, with the exception of MHR that tended to have a stronger correlation with CIMT (*r* = 0.126, *p* = 0.005) (Table 2 and Figure 2). After adjustment for confounding risk factors, MHR remained correlated significantly with CIMT in diabetic (*r* = 0.172, *p* = 0.001) but not non-diabetic (*r* = 0.006, *p* = 0.813) patients (Table 2). Moreover, we found an upward trend in the distribution of CIMT with increasing quartiles of MHR in both groups (Figure 3). The average (Figure 3A) and maximum CIMT (Figure 3B) in the fourth quartile of MHR (≥13.27) were higher than those in the first two quartiles (<10.13) in T2DM patients and those in the first quartile (<7.52) in non-diabetic subjects. Additionally, previous reports demonstrate that men have greater CIMT than women (20), so we sex-stratified our analyses to evaluate the association of MHR with CIMT using linear regression models. After accounting for age in Model 1, MHR was associated with CIMT in males but not in females both in the diabetic and non-diabetic population. After adjustment for other confounding risk factors in Model 2, MHR persisted to be associated with CIMT in males with diabetes but not in those without diabetes (Table 3).

**Table 2:** Correlation analyses for CIMT and MHR in diabetic and non-diabetic subjects.

	**CIMT**			
	**non-DM**		**DM**	
	***r***	***P*-value**	***r***	***P*-value**
Age	0.197	<0.001	0.216	<0.001
Systolic BP	0.080	0.001	0.070	0.120
Diastolic BP	0.043	0.062	0.009	0.848
BMI	0.066	0.005	0.004	0.930
Monocytes	0.023	0.329	0.088	0.050
Log hsCRP	0.056	0.019	0.001	0.981
eGFR	−0.044	0.069	−0.130	0.005
Log HbA1C	0.133	<0.001	−0.026	0.568
Log triglyceride	0.044	0.061	0.063	0.159
Total cholesterol	0.043	0.065	0.008	0.862
HDL cholesterol	−0.060	0.010	−0.079	0.078
LDL cholesterol	0.068	0.004	0.042	0.352
non-HDL cholesterol	0.063	0.007	0.029	0.521
Apolipoprotein A-I	−0.050	0.032	−0.051	0.256
Apolipoprotein B	0.070	0.003	0.029	0.516
Log MHR	0.058	0.012	0.126	0.005
Log MHR adjusted^*^	0.006	0.813	0.172	0.001

* *After adjustment for age, sex, history of smoking, systolic blood pressure, body mass index, high sensitivity CRP, eGFR, HbA1C, and LDL cholesterol*.

**Table 3:** Sex stratified associations of MHR with CIMT in diabetic and non-diabetic patients.

	**Model 1**						**Model 2**					
	**Males**			**Females**			**Males**			**Females**		
	**Sβ**	***R*^**2**^**	***P*-value**	**Sβ**	***R*^**2**^**	***P*-value**	**Sβ**	***R*^**2**^**	***P*-value**	**Sβ**	***R*^**2**^**	***P*-value**
**non-DM**	0.071	0.062	0.024	0.011	0.027	0.737	0.029	0.083	0.416	−0.006	0.040	0.878
**DM**	0.153	0.081	0.009	0.066	0.030	0.323	0.221	0.089	0.001	0.089	0.036	0.256

*R^2^, adjusted R-squared, Sβ, standardized β coefficient*.

*Model 1, age-adjusted associations*.

*Model 2, after adjusting for sex, history of smoking, systolic blood pressure, body mass index, high sensitivity CRP, eGFR, HbA1C, and LDL cholesterol*.

#### Logistic Regression Analyses for Elevated CIMT in Diabetic and Non-diabetic Subjects

**Figure 4:**
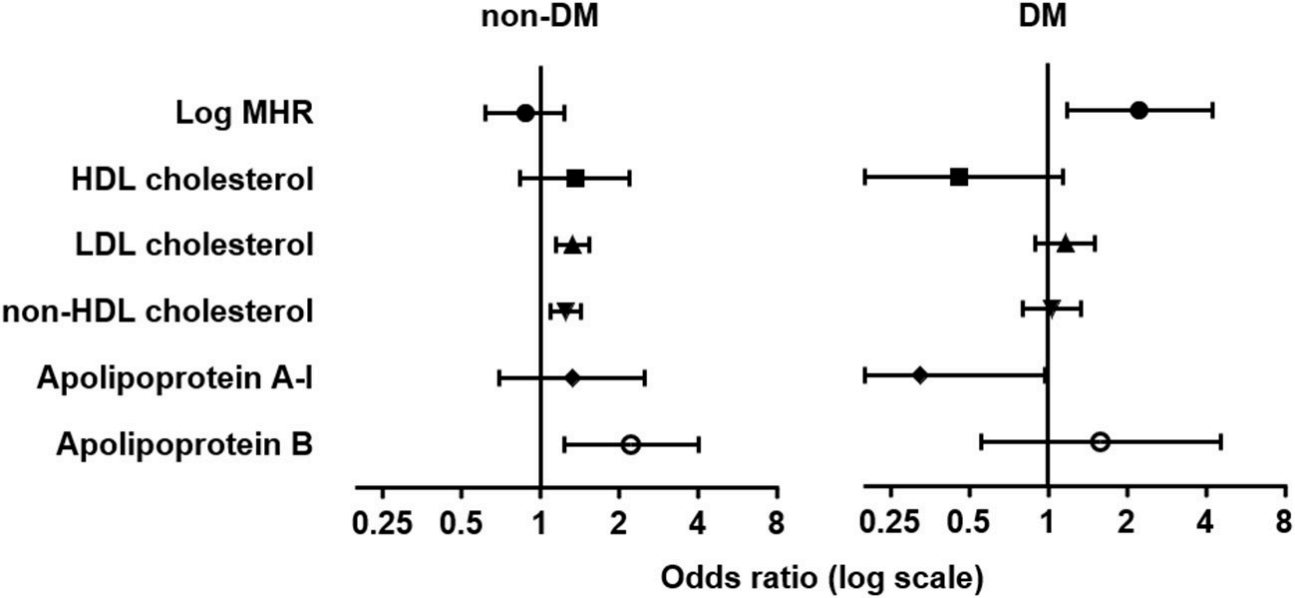
Forest plots of odds ratio (OR) and 95% confidence interval (CI) for elevated carotid intima-media thickness. Forest plots were grouped by MHR and traditional lipid variables in the adjusted logistic regression model (model 3) in non-diabetic (non-DM, left) and patients with type 2 diabetes (DM, right). Elevated CIMT was defined as CIMT in the fourth quartile (≥0.73 mm).

We then analyzed the association between elevated CIMT and different lipid parameters with logistic regression in three models (Table 4). Levels of LDL-C, non-HDL-C, and apoB were associated with elevated CIMT either in univariate analysis (model 1) or after adjusting for age, sex, history of hypertension and smoking (model 2), or with further adjustment for eGFR, log-transformed levels of hsCRP, HbA1c, and triglyceride (model 3). However, these associations were markedly attenuated in patients with T2DM. In contrast, we found log-transformed MHR was associated with elevated CIMT either in unadjusted [odds ratio (OR): 1.886, (95% CI: 1.144–3.107), *p* = 0.013] or adjusted analyses [OR: 1.756 (95% CI: 1.030–2.993), *p* = 0.038 in model 2; OR: 2.237 (95% CI: 1.172–4.270), *p* = 0.015 in model 3] in diabetic but not non-diabetic subjects (Table 4 and Figure 4).

**Table 4:** Logistic regression analyses for elevated CIMT in diabetic and non-diabetic subjects.

	**non-DM**		**DM**	
**Model**	**OR (95% CI)**	***P*-value**	**OR (95% CI)**	***P*-value**
**LOG MHR**				
Model 1	1.133 (0.878–1.463)	0.337	1.886 (1.144–3.107)	0.013
Model 2	1.070 (0.799–1.431)	0.651	1.756 (1.030–2.993)	0.038
Model 3	0.876 (0.619–1.238)	0.453	2.237 (1.172–4.270)	0.015
**HDL CHOLESTEROL**				
Model 1	0.964 (0.670–1.388)	0.845	0.522 (0.262–1.039)	0.064
Model 2	0.951 (0.636–1.421)	0.805	0.541 (0.249–1.060)	0.071
Model 3	1.351 (0.832–2.193)	0.224	0.454 (0.182–1.133)	0.091
**LDL CHOLESTEROL**				
Model 1	1.211 (1.064–1.379)	0.004	1.093 (0.883–1.352)	0.416
Model 2	1.335 (1.165–1.531)	<0.001	1.193 (0.955–1.489)	0.120
Model 3	1.325 (1.140–1.539)	<0.001	1.166 (0.897–1.516)	0.252
**non-HDL CHOLESTEROL**				
Model 1	1.154 (1.035–1.286)	0.010	1.040 (0.876–1.234)	0.657
Model 2	1.253 (1.117–1.404)	<0.001	1.134 (0.948–1.357)	0.170
Model 3	1.252 (1.091–1.435)	0.001	1.034 (0.803–1.332)	0.795
**APOLIPOPROTEIN A-I**				
Model 1	1.014 (0.598–1.719)	0.960	0.502 (0.205–1.229)	0.131
Model 2	1.023 (0.569–1.841)	0.939	0.558 (0.217–1.437)	0.227
Model 3	1.322 (0.696–2.511)	0.394	0.326 (0.108–0.977)	0.045
**APOLIPOPROTEIN B**				
Model 1	1.821 (1.123–2.953)	0.015	1.192 (0.541–2.625)	0.662
Model 2	2.410 (1.455–3.992)	0.001	1.726 (0.756–3.939)	0.195
Model 3	2.225 (1.233–4.015)	0.008	1.587 (0.555–4.540)	0.389

*Model 1 is unadjusted model; Model 2 is adjusted for age, sex, history of hypertension and smoking; Model 3 is adjusted for age, sex, history of hypertension and smoking, eGFR, logarithmically transformed levels of C-reactive protein, HbA1c and triglyceride*.

### Study 2

#### Baseline Characteristics of the Diabetic Cohort

Based on these findings from study 1, we propose that MHR is a useful assessment in prediction of subclinical atherosclerosis, as reflected by thickening of CIMT, in patients with T2DM. To evaluate the predictive value of MHR on the progression of CIMT in diabetic patients, 110 diabetic patients with normal CIMT (<0.73 mm), and a follow-up ultrasonography at 12 months were enrolled in study 2 (Table 5). Among the recruited subjects, 66.4% patients were male, 30.0% had smoking habits, and 53.6% were comorbid with hypertension. The mean age, BMI, HDL-C, and LDL-C at baseline was 52.86 ± 10.32 years, 25.95 ± 4.64 kg/m^2^, 1.08 ± 0.32 mmol/L, and 2.70 ± 0.92 mmol/L, respectively. The medial basal MHR was 11.00 (IQR: 8.12–13.68). A total of 70% of patients were on oral hypoglycemic drugs, and 48.2% were on insulin therapy.

**Table 5:** Baseline characteristics of the diabetic cohort.

Number	110
Male, *n* (%)	73 (66.4)
Age, years	52.86 ± 10.32
Body mass index, kg/m^2^	25.95 ± 4.64
Smoking, *n* (%)	33 (30.0)
Hypertension, *n* (%)	59 (53.6)
Systolic blood pressure, mmHg	133.66 ± 18.93
Diastolic blood pressure, mmHg	77.34 ± 12.77
White blood cell (10^9^/mL)	6.37 ± 2.06
Neutrophil (10^9^/mL)	3.82 ± 1.42
Lymphocyte (10^9^/mL)	2.02 ± 0.63
Monocyte (10^9^/mL)	0.44 ± 0.13
Platelet (10^9^/mL)	186.38 ± 53.06
hsCRP, mg/L	0.77 (0.46–1.59)
Serum creatinine, μmol/L	72.24 ± 16.60
Blood urea nitrogen, mmol/L	5.32 ± 1.59
Uric acid, μmol/L	330.26 ± 81.29
eGFR, mL/min/1.73 m^2^	124.06 ± 22.56
HbA1c, %	7.60 (6.10–9.30)
Fasting glucose, mmol/L	7.31 (5.65–10.68)
Postprandial glucose (2 h), mmol/L	15.13 (10.46–18.71)
Fasting insulin, μIU/mL	8.45 (4.31–15.12)
Postprandial insulin (2 h), μIU/mL	38.31 (23.39–62.21)
Triglyceride, mmol/L	1.87 (1.19–2.60)
Total cholesterol, mmol/L	4.52 ± 1.16
HDL cholesterol, mmol/L	1.08 ± 0.32
LDL cholesterol, mmol/L	2.70 ± 0.92
Apolipoprotein A, g/L	1.28 ± 0.24
Apolipoprotein B, g/L	0.89 ± 0.25
MHR	11.00 (8.12–13.68)
Oral hypoglycemic drugs, *n* (%)	77 (70.0)
Insulin, *n* (%)	53 (48.2)

#### Correlation Between Change in CIMT and Baseline Clinical Variables

**Figure 5:**
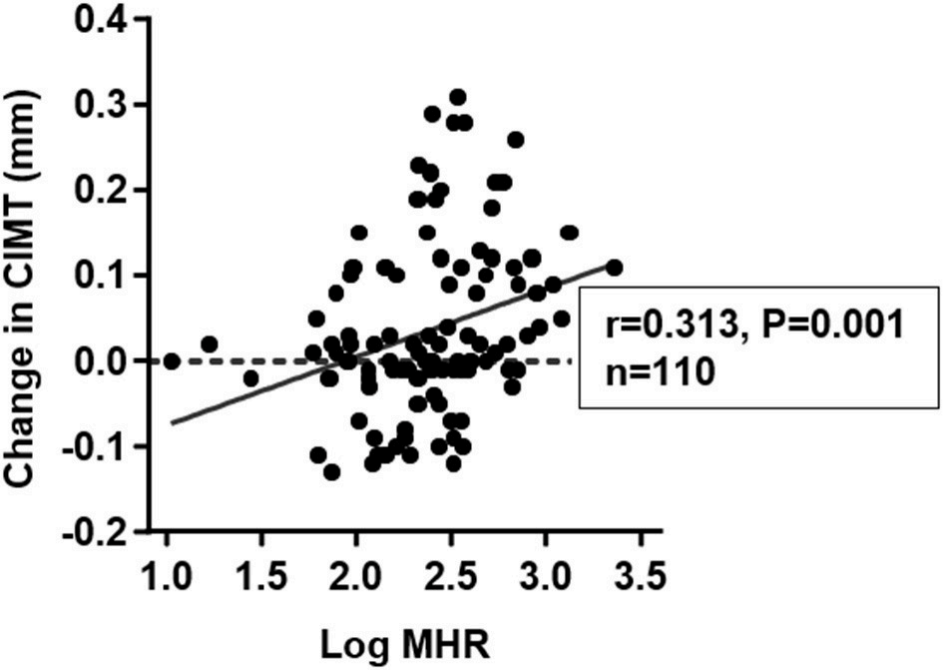
Correlation between change in carotid intima-media thickness (CIMT) and monocyte to HDL cholesterol ratio (MHR) at baseline. Basal MHR was logarithmically transformed before plotting.

Carotid ultrasonography was performed again at 1 year follow-up. The mean value of changes in CIMT was 0.037 mm. Progression of CIMT was detected in 58 (52.7%) of the subjects. The change in CIMT was positively correlated with count of monocytes (*r* = 0.350, *p* < 0.001) and log-transformed MHR (*r* = 0.313, *p* = 0.001) at baseline (Table 6 and Figure 5). There were also borderline significant associations of change in CIMT with BMI (*r* = 0.180, *p* = 0.064) and log-transformed HbA1C (*r* = 0.176, *p* = 0.078). After adjustment for confounding risk factors including age, sex, BMI, levels of hsCRP, eGFR, HbA1C, LDL-C, and history of hypertension and smoking, there was a positive but non-significant correlation between log-transformed MHR and change in CIMT (*r* = 0.223, *p* = 0.079). No significant correlation was found between change in CIMT and age, eGFR, log-transformed hsCRP, and traditional lipid parameters (Table 6).

**Table 6:** Correlation between change in CIMT and baseline clinical variables.

	**Chang in CIMT**	
	***r***	***P*-value**
Age	0.059	0.541
BMI	0.180	0.064
Monocytes	0.350	<0.001
Log hsCRP	0.055	0.621
eGFR	−0.090	0.368
Log HbA1C	0.176	0.078
Log triglyceride	−0.004	0.968
HDL cholesterol	−0.093	0.335
LDL cholesterol	0.009	0.925
non-HDL cholesterol	−0.016	0.871
Apolipoprotein A-I	−0.132	0.172
Apolipoprotein B	0.051	0.595
Log MHR	0.313	0.001
Log MHR adjusted^*^	0.223	0.079

* *After adjustment for age, sex, history of smoking, hypertension, body mass index, high sensitivity CRP, eGFR, HbA1C, and LDL cholesterol*.

#### Multivariate Linear Regression Analyses for Change in CIMT

In multivariate linear regression analysis (Table 7), we found male gender, age, history of hypertension, and baseline CIMT were independently associated with change in CIMT (model I). When log-transformed MHR was included in the model, it remained to be an independent determinant of change in CIMT [β = 0.059, (95% CI: 0.013–0.105), *p* = 0.012] (model II). The inclusion of MHR resulted in an improvement in the predictive ability of the model (change in *R*^2^ = 0.038, *p* = 0.012). In addition, this association was not affected by controlling for hypoglycemic therapies [β = 0.059, (95% CI: 0.012–0.105), *p* = 0.014] (model III).

**Table 7:** Multivariate linear regression analyses for change in CIMT.

	**Regression coefficient (95% CI)**	**Sβ**	***P*-value**	**Regression coefficient (95% CI)**	**Sβ**	***P*-value**	**Regression coefficient (95% CI)**	**Sβ**	***P*-value**
	**Model I**			**Model II**			**Model III**		
Male gender	0.046 (0.007–0.084)	0.218	0.021	0.031 (−0.008–0.070)	0.148	0.121	0.031 (−0.009–0.071)	0.146	0.130
Ages (per 10 years)	0.021 (0.004–0.038)	0.216	0.015	0.020 (0.004–0.037)	0.208	0.017	0.019 (0.001–0.036)	0.191	0.037
Hypertension	0.043 (0.010–0.076)	0.222	0.011	0.040 (0.008–0.072)	0.206	0.016	0.040 (0.008–0.073)	0.209	0.016
Smoking	0.015 (−0.024–0.055)	0.071	0.441	0.012 (−0.027–0.050)	0.054	0.550	0.012 (−0.027–0.051)	0.057	0.532
Log HbA1C	0.059 (−0.013–0.130)	0.137	0.106	0.062 (−0.007–0.132)	0.145	0.079	0.044 (−0.044–0.133)	0.104	0.322
Log triglyceride	−0.008 (−0.036–0.020)	−0.046	0.583	−0.020 (−0.049–0.009)	0.117	0.180	−0.018 (−0.048–0.012)	−0.108	0.237
Baseline CIMT	−0.809 (−1.099–0.518)	−0.477	< 0.001	−0.761 (−1.046–0.475)	0.449	< 0.001	−0.748 (−1.041−0.454)	−0.441	0.000
Log MHR	–	–	–	0.059 (0.013–0.105)	0.234	0.012	0.059 (0.012–0.105)	0.232	0.014
OHA							−0.004 (−0.041–0.034)	−0.018	0.848
Insulin							0.015 (−0.028–0.058)	0.074	0.496
	Adjusted *R*^2^ = 0.275			Adjusted *R*^2^ = 0.313			Adjusted *R*^2^ = 0.302		

*Sβ, standardized regression coefficient β*.

*OHA, oral hypoglycemic agent*.

## Discussion

The major findings of the present study are that correlation between MHR and CIMT is enhanced in patients with T2DM than non-diabetic subjects. MHR is superior to traditional lipid variables in association with CIMT thickening and is an independent predictor of the progression of CIMT in patients with T2DM.

MHR appears to be a novel and convenient maker with integration of pro-inflammatory and anti-inflammatory indices. In this study, we demonstrate a more prominent role for MHR in prediction of subclinical carotid atherosclerosis in diabetic than non-diabetic populations. First, correlations with CIMT were comparable between MHR and traditional lipid parameters including HDL-C, LDL-C, non-HDL-C, apoA-I, and apoB in non-diabetic subjects. In patients with T2DM, a stronger correlation was observed between CIMT and MHR, whereas those with other lipid variables tended to be attenuated. Second, we detected an upward trend in the distribution of CIMT with increasing quartiles of MHR in both groups. Especially, CIMT in the fourth quartile of MHR was significantly higher than that in the first two quartiles in diabetic patients, and also higher than that in the fourth quartile of MHR in non-diabetic patients. Third, in the logistic regression analyses, elevated CIMT was independently associated with LDL-C, non-HDL-C, and apoB, whereas these associations were markedly attenuated in the diabetic population. Interestingly, we found MHR was independently associated with elevated CIMT both in unadjusted and adjusted models in the diabetic population, while such association was no longer significant in non-diabetic subjects. Finally, in study 2, we further showed that baseline MHR has greater correlation than traditional lipid parameters with change in CIMT at 1 year follow-up.

Monocytes play an important role in the development of diabetic complications (21). Monocyte counts have been shown to be associated with insulin resistance, type 2 diabetes (22, 23), coronary artery disease (24), diabetic micro-, and macrovascular complications (25, 26). Previously, Matsumura et al. reported that monocyte counts were positively correlated with CIMT in patients with T2DM (10). On the other hand, emerging data suggest that low HDL-C is an important contributor to accelerated atherosclerosis in diabetic patients (27). Therefore, the integrated maker, MHR, is supposed to be a better predictive factor than each of the variables in association with vascular structural change in diabetic patients as we demonstrated in the present study. In contrast, the imbalance between pro- and anti-inflammatory mechanisms in non-diabetic conditions is generally relatively moderate, which might explain our findings that MHR is associated with thickening and progression of CIMT in diabetic but not non-diabetic subjects. Previously, Kanbay et al. reported that MHR acts as an independent predictor for cardiovascular events in patients with chronic kidney disease and was increased in parallel with decreasing eGFR (12). MHR was also correlated with flow-mediated dilation of brachial artery in patients with Behcet disease (28). Taken together, these lines of evidence imply that the predictive value of MHR for cardiovascular disease is improved in conditions of chronic inflammatory disorders such as diabetes.

Our data reveal that MHR is both associated with basal thickness and progression of CIMT in patients with T2DM. Noteworthy, while CIMT has been well-evidenced to predict the risk of the subsequent cardiovascular events (29–31), the prognosis value of CIMT rate of change in evaluating cardiovascular risk remain inconclusive (31–34). Findings from the Multi-Ethnic Study of Atherosclerosis (MESA) point to a positive association between CIMT progression and incident stroke (32). The IMPROVE study showed that the fastest maximum CIMT progression, but not other CIMT measures, was significantly associated with the subsequent vascular events (35). Conversely, meta-analyses on 16 cohorts comprised of 36,984 participants in the general population (31), or 21 cohorts comprised of 3,902 participants in patients with T2DM (33), both detected no association between CIMT progression and cardiovascular risk. Nevertheless, the thickening of CIMT, compared to those with unchanged or regressed CIMT, over the 1 year follow-up period appears to some extent reflect the adverse structural changes of the arterial wall. The prognosis value of MHR in predicting CIMT progression awaits examination in prospective trails.

We appreciate limitations in our study. First, this study was a retrospective analysis based on prospectively collected data, and all the enrolled patients were from a single center. Second, the sample size in study 2 was modest and therefore the ability to definitely evaluate the association of CIMT progression with MHR and other variables in patients with T2DM was limited. Third, MHR was not dynamically monitored, so it is still not known changes in MHR is associated with the progression of CIMT. Further prospective studies are warranted to analyze whether decreasing MHR would lead to less progression of atherosclerosis.

## Conclusions

In summary, this study suggests that elevated MHR is a convenient and effective measure in prediction of the presence and progression of subclinical carotid atherosclerosis in patients with T2DM.

## Author Contributions

JC, LL, and XW study design and conduction, data analysis, and interpretation, manuscript writing. CL, ZL, XS, and YS study conduction, data collection and manuscript revision. FD, RZ, and WS study design and manuscript revision. All authors read and approved the final manuscript.

### Conflict of Interest Statement

The authors declare that the research was conducted in the absence of any commercial or financial relationships that could be construed as a potential conflict of interest.
